# The Effect of Physical Activity on Executive Functions in the Elderly Population: A Systematic Review of Randomized Controlled Trials

**DOI:** 10.3390/brainsci15070703

**Published:** 2025-06-30

**Authors:** Carmela Matrisciano, Roberta Minino, Anna Maria Mariani, Cristiana D’Anna

**Affiliations:** 1 Department of Education and Sport Sciences, Pegaso Digital University, Centro Direzionale Isola F2, 80143 Napoli, Italy; carmela.matrisciano@univr.it (C.M.); roberta.minino@unipegaso.it (R.M.); annamaria.mariani@unipegaso.it (A.M.M.); 2 Department of Neuroscience, Biomedicine and Movement, University of Verona, 37129 Verona, Italy

**Keywords:** cognitive decline, elderly, executive functions, physical activity

## Abstract

**Background/Objectives**: In recent years, there has been increasing interest in the scientific, educational, and health sectors in investigating aspects upon which to design physical-activity interventions to prevent cognitive decline, a phenomenon that affects levels of autonomy and quality of life in older adulthood. Physical activity (PA) has been shown to be an effective strategy that can be used to preserve executive functions (EFs) by improving brain flexibility and efficiency. This systematic review aims to identify the most effective strategies used to maintain EF, prevent decline, and promote independence in the elderly. **Methods**: A systematic literature review was conducted according to PRISMA guidelines. The search was conducted in the following databases: Google Scholar, PubMed, Scopus, and Web of Science; the search used keywords such as “cognitive decline”; “cognitive flexibility”; “elderly”; “executive functions”; “inhibition”; “physical activity”; and “working memory”. Experimental studies published between 2019 and 2025 examining the effects of PA on EFs in adults over 60 were selected. After considering the inclusion and exclusion criteria, nine studies were included. The methodological quality of the included studies ranged from moderate to high according to the PEDro scale. **Results**: The analyzed studies show that short-term interventions positively affect one or two components of EFs, while medium- and long-term interventions produce benefits for all components (working memory, inhibition, and cognitive flexibility). Interventions combined with cognitive stimulation show a greater impact than PA alone. **Conclusions**: PA is an effective strategy for preserving EFs in the elderly, but the lack of standardized protocols makes it difficult to identify optimal interventions. Further research is needed to more precisely define the most effective intervention approaches.

## 1. Introduction

The current scientific literature increasingly focuses on identifying actionable factors to mitigate cognitive decline, a key component in preserving cognitive health and quality of life among older adults. In this context, physical activity stands out as a nonpharmacological intervention proven to enhance cognitive health and delay the progression of cognitive deterioration.

Executive functions encompass top–down cognitive processes essential for goal-directed behavior [1]. They also regulate the integration of internal and external stimuli, enabling the generation of context-appropriate responses [2,3]. As executive functions do not constitute a unitary construct, they are typically classified into two main categories: basic executive functions and higher-order executive functions [4].

Basic executive functions include inhibition, working memory, and cognitive flexibility [5]. Inhibition refers to the capacity to regulate attention, behavior, and thoughts by resisting internal impulses or external stimuli, thereby enabling contextually appropriate responses. Working memory is defined as the ability to temporarily store and manipulate information, a process essential for data integration and goal-oriented behavior. Cognitive flexibility refers to the ability to shift between mental perspectives, a process that requires inhibiting prior frameworks and integrating new ones within working memory [1].

Building upon these core processes, higher-order executive functions are structured, including reasoning, problem solving, and planning [6,7]. Reasoning involves transformation of implicit information into explicit representations, clarifying logical processes when required and arriving at coherent conclusions. Problem solving refers to the ability to achieve a goal through a sequence of cognitive operations, or, alternatively, by employing intuitive strategies. Planning entails the ability to organize goal-directed actions within specific contexts and to anticipate future events by sequencing intermediate steps.

Both basic and higher executive functions play crucial roles in a person’s overall health, biopsychosocial well-being and success in school and life. Many authors in their studies have focused on executive functions in both adolescents and older adults [8], as these functions develop progressively through early adulthood and subsequently decline in older age due to structural and functional changes in the prefrontal cortex [1,9].

Executive functions are fundamental to cognitive development, as they enhance adaptive responses to environmental demands and impact upon physical health, academic achievement, and cognitive, social, and psychological development [10]. The maturation of executive functions undergoes significant variation throughout childhood and adolescence [11,12]. To better understand the developmental trajectory of executive functions, reference can be made to the study by Laureys et al. (2022) [13], which suggests that, between the ages of 3 and 5, executive functions operate as a unified construct [14], likely due to the structural and functional immaturity of the prefrontal cortex [15]. Regarding the primary school years, a two-factor model of executive functioning, primarily comprising working memory and inhibition, has been observed; this model corresponds with the ongoing maturation of the prefrontal cortex. In contrast, a three-factor model, consisting of working memory, inhibition, and cognitive flexibility, describes the changes that emerge during adolescence [5].

In later adulthood, a gradual decline in cognitive functions is frequently observed [16], encompassing domains such as decision-making, problem-solving, planning, and response processing [17,18,19]. These changes may serve as early indicators of emerging neurodegenerative conditions, including vascular dementia and Alzheimer’s disease [20], as well as depressive symptoms [21] and other related impairments. Such cognitive decline negatively impacts the capacity to independently manage everyday tasks and maintain functional autonomy [22,23,24]. In this context, it is useful to distinguish between activities of daily living (ADLs) and instrumental activities of daily living (IADLs) [25]. Specifically, ADLs refer to basic self-care routines that constitute the core of an individual’s functional status (e.g., eating, bathing, and dressing). Instrumental activities of daily living (IADLs), on the other hand, involve more complex tasks that demand greater autonomy (e.g., using the telephone, taking medications, and household activities) [26]. To gain a comprehensive understanding of how executive functions influence the performance of daily living activities, it would be pertinent to refer to the work of Mlinac and Feng [27]. According to these two scholars, the ability to perform both ADLs and IADLs relies on the integration of cognitive, perceptual, and motor skills, and is inversely related to cognitive decline. This suggests that cognitive decline may adversely impact quality of life, as difficulties in executing daily routines compromise the autonomy and self-efficacy of older adults. Self-esteem refers to the perception of one’s personal worth, whereas self-efficacy is a psychosocial construct that reflects confidence in one’s abilities and in the capacity to successfully carry out tasks across various contexts [28]. Self-efficacy has been widely investigated in relation to physical functioning, physical activity, and overall quality of life [29]. In particular, physical limitations that impair the execution of daily tasks can significantly affect an individual’s sense of self-efficacy. Moreover, older people with high levels of self-efficacy demonstrate stronger belief in their own capabilities, which positively influences their ability to independently perform ADLs [30]. Conversely, individuals with low self-efficacy tend to avoid cognitively demanding tasks [31]. Furthermore, such individuals often exhibit low self-esteem, reduced subjective well-being, and, in some cases, symptoms of depression.

Quality of life, both in adulthood and old age, is a multifaceted concept influenced by various factors [32], including the perceptions of one’s health status, physical capabilities, autonomy in daily activities, and interpersonal relationships [33]. The inevitable process of aging, which defines the evolution of the life cycle, is a complex phenomenon encompassing physical, psychological, environmental, and social dimensions [34]; these interact reciprocally and are dynamic and non-linear in nature. According to Falck et al. [35], aging is associated with impairments in both physical and cognitive functions, which interact with one another [36]. A decline in cognitive ability is often correlated with a reduction in walking speed [37], and slower gait speed may serve as an early indicator of cognitive decline [38].

The evolution and subsequent decline of certain abilities are undoubtedly influenced by endogenous factors, such as genetic predisposition and biological processes associated with aging; however, exogenous factors, including lifestyle, social environment, and educational level, also contribute to shaping their trajectories. Therefore, to mitigate the risk of cognitive decline and preserve levels of independence, it is essential to adopt a healthy lifestyle [39].

In recent years, there has been increasing interest across the scientific, educational, and healthcare sectors in developing strategies to enhance cognitive abilities and mitigate cognitive decline in the elderly population. This interest is partially driven by WHO projections, which estimate that by 2030, approximately one in six individuals will be aged 60 or older [40]. Physical inactivity, defined as a reduction in bodily movement resulting in decreased energy expenditure, is a common factor in both middle age and adulthood [41]. In this context, the WHO provided guidelines on physical-activity recommendations for adults and the elderly in 2020. The guidelines recommend that both adults and older adults engage in 150–300 min of moderate physical activity or 75–150 min of vigorous activity per week. The guidelines recommend that both adults and older adults engage in 150–300 min of moderate physical activity or 75–150 min of vigorous activity per week. The WHO also defines physical activity as any bodily movement produced by skeletal muscles that results in energy expenditure. Physical activity can be performed at varying intensities and encompasses activities related to work, home, or leisure contexts, including sports, walking, cycling, recreation, and active play [42].

As stated by Rodriguez-Rodríguez et al. [43], physical activity is associated with executive functions across all age groups, from youth to older adults; moreover, it is an effective strategy [44], as it benefits both cognitive [45,46,47] and physical health [35]. Compared to individuals leading sedentary lifestyles, those who regularly engage in moderate- to high-intensity physical activity tend to experience significant improvements in various psychological and cognitive domains, including reduced anxiety, stress, and depression; higher self-esteem; improved mood; and enhanced cognitive function [48]. Physical activity aids in making the brain more efficient, flexible, and adaptive, resulting in significant enhancement of memory and executive functions. This occurs through positive changes that stimulate processes such as neurogenesis, synaptic plasticity, and neuronal growth [45,49]. Therefore, as noted by Engeroff et al. [50], Bento-Torres et al. [51], Pindus et al. [52], and Chen and Nakagawa [53], physical activity mitigates aging-related neurodegeneration and the subsequent decline in cognitive function [54]. This benefit is particularly relevant given that aging is frequently associated with cognitive decline, impacting processing speed, reasoning, memory, and executive functions.

The benefits associated with the optimal amount of exercise are influenced by factors such as the intensity, frequency, type, and duration of the activity. However, the understanding of these aspects remains unclear [55]. Liu et al. [56] in their study also highlighted the difficulty in fully understanding the relationship between physical activity and cognitive function due to the limited number of studies with large samples and the insufficient exploration of causal relationships.

Palta et al. [57] identified another challenge related to studies investigating the role of physical activity in adulthood to counteract cognitive decline. Specifically, they noted that there is an insufficient number of studies that include repeated measurements and sufficiently long follow-ups to investigate the relationship between physical activity and cognitive decline.

Several studies, including that by Erickson et al. [49], have identified aerobic exercise, resistance training, mind–body practices, and multimodal programs as effective in enhancing cognitive function in older adults, regardless of the presence of cognitive impairment. Aerobic exercises are typically performed at moderate intensity and engage large muscle groups, thereby improving cardiovascular efficiency. In contrast, resistance training involves muscle contractions against external resistance, aiming to enhance muscular strength and endurance. In recent years, increasing attention has also been directed toward mind–body practices such as yoga, tai chi, and qigong, in addition to traditional aerobic training [58]. These practices integrate stretching, relaxation, and meditation components, and have been shown to provide significant benefits for executive functioning [58,59]. Tai chi is a multifaceted discipline that combines elements of aerobic and anaerobic exercise with flexibility training [7,60]. Qigong consists of a series of static or dynamic exercises that integrate controlled breathing with coordinated body movements, aiming to harmonize internal energy and promote overall physical well-being. Yoga, meanwhile, involves the practice of physical postures (asanas), breathing techniques (pranayama), and meditation, with the objective of fostering physical equilibrium and mental clarity [7,60].

Considering the findings discussed thus far, this review aims to further explore the relationship between physical activity and executive functions, with particular emphasis on adulthood and later life. The central hypothesis of this review posits that regular physical activity exerts a positive influence on executive functions and may help attenuate cognitive decline in later life.

Through a critical examination of the scientific literature and a comparative analysis of various physical-activity protocols, this review seeks to identify the most effective strategies for preserving executive functions and mitigating cognitive decline, thereby promoting the maintenance of autonomy and independence in daily living activities.

## 2. Materials and Methods

### 2.1. Literature Search

A literature search was conducted across the following databases: Google Scholar, PubMed, Scopus, and Web of Science; the search was performed in accordance with the PRISMA guidelines [61].

The protocol of this systematic review was retrospectively registered in the Open Science Framework (OSF): https://osf.io/5fj6y/, accessed on 3 June 2025.

The search was conducted from 1 November 2024 to 28 February 2025, with the objective of synthesizing current knowledge on the relationship between physical activity and executive functions through an analysis of studies published between 2019 and 2025.

The following keywords and their combinations were used in the search: “adulthood”, “cognitive decline”, “cognitive flexibility”, “cognitive functions”, “elderly”, “executive functions”, “inhibition”, “physical activity”, and “working memory”.

The following search strings were applied: [(executive functions) or (inhibition) or (working memory) or (cognitive flexibility) and (cognitive decline) and (adulthood) and (elderly)] and [(executive functions) or (cognitive functions) and (cognitive decline) and (elderly)].

In addition, the reference lists of the included studies were manually reviewed to identify any additional relevant articles.

### 2.2. Study Selection and Screening Process

The systematic review approach was selected in order to identify and synthesize the existing scientific literature on a complex and multidimensional subject, with the aim of highlighting the key aspects related to the topic under investigation. Specifically, studies were eligible for inclusion in this literature review if they met the following criteria:

Type of study: Experimental.

Language: English. (No translation procedures were required.)

Participants: Those of adult age.

Interventions: Physical activity and/or cognitive activity as a tool that stimulates executive functions.

Results: Effects of physical activity on executive functions.

In contrast, the following were excluded: literature reviews; studies in which the participants were younger than 60 years old; and studies that did not focus on executive functions.

### 2.3. Study Procedures

The literature search was carried out by two reviewers, who selected studies according to predefined criteria by evaluating titles and abstracts. Two reviewers independently screened titles and abstracts, and disagreements were resolved through discussion. Subsequently, all authors independently reviewed the selected full texts to assess their final eligibility based on the inclusion and exclusion criteria. Furthermore, the reference lists of the selected studies were examined to identify additional studies for inclusion in this review.

In the studies included in this systematic review, various tests assessing executive function were conducted both before and after the physical-activity intervention. All studies yielded consistent results, except for one [25] that reported no significant differences.

The 9 studies included in this systematic review were independently assessed by each author, and each study was found to exhibit a robust methodological design and employ standardized instruments and structured intervention protocols, ensuring the reliability of the results. However, methodological limitations are present due to the absence of an active control group [25,62,63,64,65,66]. This factor introduces a potential risk of bias that may influence the generalizability of the results. Furthermore, the results of each study were analyzed regarding the impact of physical activity on executive functions.

The methodological quality of the included studies was assessed using the PEDro scale (Table 1), which covers key domains of internal validity such as random allocation, concealed allocation, baseline comparability, blinding, follow-up, and data analysis. The potential risk of reporting bias due to missing results was taken into account when interpreting the findings, although no formal statistical analysis (e.g., funnel plot) was conducted.

For each study included in the review, the following information was considered: the source (author and year of publication), sample characteristics (number of participants and mean age), research objectives, methodological approach, type of physical activity examined, study duration, assessment tools employed, key results obtained, and PEDro score.

## 3. Results

**Scheme 1:**
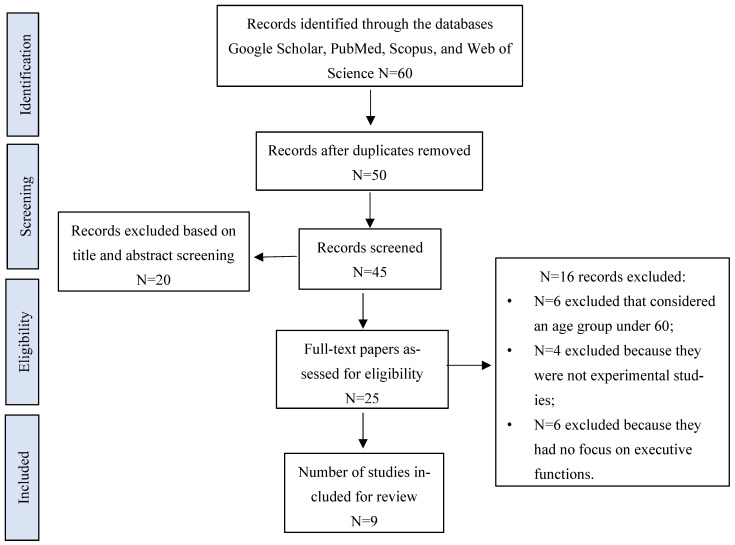
PRISMA flow diagram of the study selection process. Search conducted for articles published between 1 November 2024 and 28 February 2025.

Scheme 1 illustrates the process of the literature review, which comprised several steps. Initially, 60 articles were identified through searches in Google Scholar, PubMed, Scopus, and Web of Science databases. After the removal of duplicates, the number of articles was reduced to 50. Of these, 45 articles were screened based on title and abstract, which resulted in the exclusion of 20 studies. After this screening, 45 articles were subjected to further evaluation and 25 were considered suitable for full-text analysis. However, 16 articles were excluded for the following reasons:

Six articles included a sample with an age below 60 years,

Four articles were not experimental studies,

Six articles did not report any effects on executive functions.

Finally, the total number of studies included in the systematic review amounted to nine.

The data from each study included in this systematic review were processed and are summarized in Table 1. Specifically, for each study, the table presents information on the source (authors), sample (sample size, mean age of participants), objective, methodology, type of physical activity, duration, assessment tools, key results obtained, and PEDro score.

Quantitative details of the physical-activity interventions (e.g., intensity, frequency, and duration) are available in Supplementary Table S1. Baseline characteristics of the participants are reported in Supplementary Table S2. The types and difficulty levels of cognitive training tasks are described in Supplementary Table S3.

In light of the findings reported thus far, physical activity appears to be an effective strategy for preserving and enhancing executive functions, one that counteracts cognitive decline in both adults and the elderly. Among the detailed studies, several reported positive effects on all three components of executive functions [65,66,67,68,69], one on inhibition [63], one on inhibition and working memory [62], and one on flexibility and inhibition [64]; only one study reported a lack of significant effects [25].

To facilitate the analysis of the studies reviewed, they were organized according to a progressive criterion based on the duration of the intervention.

Specifically, in studies that implemented short-duration interventions, positive effects were observed on some components of executive function. Therefore, even a brief session of physical activity can have a positive impact on executive functions, although the effects may be limited [62]. In the same study, a single 20 min session of moderate-intensity aerobic exercise was found to improve inhibition and certain aspects of working memory in elderly individuals. The objective was to determine whether 20 min of exercise could positively impact specific cognitive functions, such as working memory and the ability to control impulsive responses. Participants were assigned to an experimental group, which engaged in 20 min of cycling, or a control group, which remained inactive. Both groups completed four cognitive tests, both before and after the intervention: the Affective Go/No-Go (AGN), Simple Reaction Time (SRT), Spatial Working Memory (SWM), and a backward counting task. Upon completion, neurocognitive assessments revealed improvements in inhibitory control functions and certain aspects of working memory in the experimental group. Similar positive effects were observed by Nouchi et al. [63], who evaluated the beneficial impacts of 30 min of physical training on both cognitive and emotional functions in women with a mean age of 62.29. The sample was divided into an exercise group that engaged in aerobic, strength-based, and stretching exercises, and a control group that did not participate in any exercise. Cognitive functions were assessed both before and after the intervention in both the control and exercise groups using the following tests: the Wechsler Adult Intelligence Scale, the Stroop Task, the reverse Stroop Task, and a verbal fluency task. The results indicated that the combined exercise group exhibited improvements in executive function inhibition when compared to the control group.

The results that emerged from the study by Lebeau et al. [64] corroborate the conclusions of Chen et al. [4], Lin et al. [70], and Xiong et al. [71], namely, that aerobic exercise can enhance cognitive flexibility in healthy middle-aged and elderly adults. Specifically, Lebeau et al. [64] divided the sample into two groups: an exercise group (which cycled on a cycloergometer at moderate intensity for 20 min) and a control group (which watched a 25 min informational driving video). The following instruments were employed: the Trail-Making Test, the Stroop Test, and the Useful Field of View Test. After the intervention, the exercise group demonstrated significant improvements in tests of mental flexibility (Trail-Making Test) and in the inhibition of automatic responses (Stroop Test). Lebeau et al. [25] investigated the impact of a 20 min cycling session on executive function, balance, and activities of daily living in 62 healthy elderly individuals. Participants were randomly assigned to two groups: an exercise group and a control group. Members of the exercise group performed 20 min of cycling, while the control group watched a video on the importance of exercise and rest for the well-being of the elderly population. Cognitive assessments, including the Mini Mental State Examination, the Geriatric Depression Scale, tests for color blindness, and the Stroop Test, were conducted following the intervention. No significant differences were found between the two groups, suggesting that factors such as the type of exercise and its intensity, as well as the experimental context, may influence the results.

The findings from these studies are particularly relevant, as inhibition is a crucial executive function that influences both attention and behavior, two key factors in reducing the risk of falls [25].

After investigating the findings in the short-term studies, the study turned its focus to the effects of physical activity on executive functions as described in the medium- and long-term studies.

Specifically, improvements in the three executive functions have been reported in medium-term (12-week) and long-term (3–6 months) studies. For instance, Byun et al. [67], Pellegrini-Laplagne et al. [68], and Pereira et al. [65] implemented three-month interventions and Díaz-García et al. [66] conducted a two-month intervention, while Gervasi et al. [69] implemented a six-month intervention.

Byun et al. [67] investigated the impact of light exercise on executive function in 81 middle-aged and older adults. The experimental group engaged in light cycling exercises three times a week for 30–50 min, while the control group maintained their usual lifestyle habits. Both groups completed the Stroop Test before and after the intervention. Notably, the experimental group showed improved performance, suggesting enhanced executive function. These results align with the findings of Morris et al. [72], who argue that light physical activity enhances executive functions, particularly by promoting the efficient activation of the prefrontal cortex, which is crucial for tasks requiring concentration and decision-making skills.

The pilot study conducted by Pellegrini-Laplagne et al. [68], involving 35 elderly participants, aimed to compare the effects of combined exercise training and cognitive stimulation with those of isolated interventions. The sample was divided into three groups: physical training, cognitive training, and combined exercise and cognitive training. The combined group participated in two 30 min sessions per week for 12 weeks. Neuropsychological assessments were conducted both before and after the intervention, and the following tests were administered: Montreal Cognitive Assessment (MOCA), 2-back, Stroop, Trail Making, Rey Auditory Verbal Learning Test (RAVLT), Digit Symbol Substitution, and Baroreflex Sensitivity (BRS). All groups showed improvements in executive performance, including flexibility and working memory. The combined group, however, demonstrated significant improvements in inhibitory control.

In their study, Pereira et al. [65] implemented a personalized protocol combining outdoor aerobic physical activity with laboratory sessions, using a technological platform. Participants were assigned to two groups: the control group (no activity) and the intervention group (physical activity and functional exercises). Cognitive function was assessed using the Cambridge Neuropsychological Test Automated Battery platform at both the baseline and three months after the intervention, in both groups. Based on the study’s conclusions, it can be inferred that personalized physical activity is an effective strategy for counteracting age-related cognitive decline, as it allows for the implementation of activities tailored to individual needs, leading to significant improvements, both physically and cognitively.

Díaz-García et al. [66] also explored the effects of both cognitive and physical training on physical and cognitive outcomes in 24 sedentary women, who were divided into three groups: one receiving both physical and cognitive activity, one with physical activity only, and one control group. Evaluations conducted at three time points (ex ante, in itinere, and ex post), using the Brief Stroop Task and Brief Psychomotor Vigilance Task, evidenced that combined training is the most effective in improving cognitive and physical function.

Finally, Gervasi et al. [69] examined the relationship between physical activity, cognitive function, and social well-being in a sample of 68 elderly individuals aged 65 to 83 years. Participants were randomly assigned to two groups. Both groups followed the same program, which included adapted physical activity as well as cognitive and social-emotional training. Cognitive function was assessed both before and after the intervention using the following tests: the Short Form Health Survey, the Trail Making Test, and the Serial Repetition Bisyllabic Words Test. The results confirmed that physical activity positively impacts both cognitive function and social well-being.

The methodological quality of the included studies, as assessed by the PEDro scale, ranged from 4 to 7, indicating an overall moderate to high quality.

## 4. Discussion

### 4.1. Summary of Main Findings

Considering the findings discussed, the studies confirm that physical activity is an effective tool for improving executive functions and counteracting cognitive decline. Interventions of varying durations, ranging from two to six months, have shown significant benefits in terms of cognitive flexibility, working memory, inhibitory control, and social well-being.

Based on the analysis of studies examining the duration of physical-activity interventions, it can be inferred that physical activity is an effective tool for promoting cognitive well-being. Furthermore, the results emphasize that brief exercise sessions can be easily incorporated into the daily routines of elderly individuals, significantly enhancing their quality of life.

Studies of medium [65,66,67,68] and long duration [69] have shown that physical activity improves executive functions. Specifically, the results of Byun et al. [67] align with those of Morris et al. [72], who argue that light physical activity enhances executive functions, particularly by promoting the efficient activation of the prefrontal cortex, which is essential for tasks requiring concentration and decision-making abilities. The conclusions of Pereira et al. [65] suggest that personalized physical activity is an effective tool for mitigating age-related cognitive decline, as it enables the implementation of activities tailored to individual needs, resulting in significant improvements, both physically and cognitively.

The analyzed data emphasize that adopting an active lifestyle contributes to the preservation of cognitive abilities and the enhancement of quality of life in older adults, while also promoting mental and physical well-being, as well as social engagement.

Following the analysis of the studies based on their duration (short, medium, and long) and their contribution to determining the relationship between physical activity and cognitive decline, it is essential to focus on the protocols employed in the studies included in this review. Specifically, three studies incorporated a combination of physical and cognitive training [66,68,69], while six focused exclusively on physical activity [25,62,63,64,65,67]. Pellegrini-Laplagne et al. [68], Díaz-García et al. [66], and Gervasi et al. [69] demonstrated in their studies that the combination of physical training and cognitive stimulation resulted in significant improvements, compared to physical or cognitive interventions alone. One possible explanation for the effectiveness of combining physical and cognitive activities is that physical training enhances brain plasticity by stimulating cell growth and strengthening synaptic connections, while cognitive exercise specifically targets this plasticity in brain regions associated with mental tasks, expanding the number of neurons within existing neural networks [73].

Another crucial consideration in the discussion of physical activity and cognitive decline in older adulthood involves the psychological and social factors that impact the health of elderly individuals. Recent studies have demonstrated that cognitive decline may render elderly individuals more susceptible to loneliness, and this relationship appears to be bidirectional. Specifically, some research suggests that loneliness may increase the risk of both physical frailty and cognitive decline [74]. As a result, participating in physical activity, particularly in group settings, fosters the development of interpersonal relationships and provides a sense of belonging, and thus mitigates feelings of loneliness while promoting overall well-being [75].

The current scientific research has placed significant emphasis on neuroimaging studies that highlight how the regional characteristics of the aging brain can benefit from exercise. These studies demonstrate that physical activity enhances executive function and inhibitory control in older adults [76]. Executive function encompasses several regions within the frontal and parietal lobes, including the medial prefrontal cortex, superior and middle frontal gyri, precuneus, and parietal lobules [77,78]. As individuals age, there is a reduction in gray matter volume, particularly in the frontal regions and the default mode network, that negatively impacts the activation of critical regions during cognitive tasks [79,80].

Physical activity has been shown to be particularly effective in modulating brain activity within these regions, with age-dependent variations. In their meta-analysis, Yu et al. [81] noted that, in older adults, exercise stimulates the parietal cortex and the default mode network (DMN), whereas in young adults, its effects are most pronounced in the precuneus and posterior cingulate cortex. Additionally, physical activity promotes the activation of structures such as the superior frontal gyrus and precuneus, while concurrently reducing activity in specific regions of the DMN, thereby strengthening brain networks crucial for inhibitory control.

In their study, Byun et al. [67] employed functional near-infrared spectroscopy (fNIRS), a neuroimaging technique, to assess brain activity during the Stroop Test, focusing on changes in the activation of the lateral prefrontal cortex in response to Stroop interference. The study aimed to examine how moderate exercise influences this brain region in older adults by investigating alterations in neural substrates in the context of neural efficiency. The findings indicated that light exercise does not directly increase brain activation; however, it may enhance neural efficiency in the elderly, facilitating more efficient cognitive processing with reduced resource expenditure.

### 4.2. Methodological Quality and Risk of Bias

The methodological quality of the included studies, assessed using the PEDro scale, ranged from 4 to 7. Most studies scored between 5 [25,62,66,67,68,69] and 6 [64], reflecting adequate randomization and complete outcome reporting, but with limited methodological rigor due to lack of blinding and absence of intention-to-treat analyses.

One study [63] received a PEDro score of 7, standing out for including both blinded outcome assessment and an intention-to-treat approach—two key elements that reduce bias and enhance internal validity.

Studies scoring 4/10 [65] lacked randomization and blinding, increasing the risk of bias; nonetheless, they reported complete datasets and valid statistical analyses, making them informative, albeit less methodologically robust.

These findings are summarized in Table 1. Notably, studies of higher methodological quality (i.e., PEDro ≥ 6) tended to report more consistent improvements in executive functions, suggesting a potential association between study design and observed cognitive benefits.

### 4.3. Heterogeneity of Assessment Instruments

The studies included in this systematic review used a variety of cognitive tasks to assess executive functions, such as the Stroop Test, the Trail Making Test, the n-back, and Digit Span. Although each of these instruments targets a component of executive functions, they differ in psychometric properties and sensitivity in detecting changes following physical-activity interventions. This methodological heterogeneity may partly explain the variability in results. For example, the Stroop Test is widely considered the “gold standard” for measuring inhibitory control, while other instruments may be less sensitive or more susceptible to the effects of practice.

Another key point of discussion concerns the neuropsychological instruments used repeatedly in the studies included in this systematic review. In this case, the Stroop Test, Trail Making Test, and n-back are instruments that allow the investigation of different components of executive functions. Specifically, the Stroop Test is one of the most established instruments for assessing cognitive inhibition. It requires naming the color of the ink of incongruent words (e.g., the word “red” written in blue), activating a conflict between automatic reading and the required response. This interference effect makes it particularly sensitive to changes in executive functions and is therefore considered a “gold standard” for measuring inhibition.

The Trail Making Test is commonly used to assess cognitive flexibility, as it requires sequentially alternating numbers and letters under time constraints. This ability to switch quickly between different cognitive sets reflects a key component of executive functions. The n-back is commonly used to assess working memory, as it requires the constant monitoring, maintenance, and updating of a series of stimuli over time. Participants must identify the point at which a stimulus repeats itself after n positions, which involves central processes of working memory. This variability in assessment tools and their respective sensitivities may have contributed to the differences in results observed between studies, representing a potential source of heterogeneity.

### 4.4. Intervention Parameters

Regarding the characteristics of the intervention, we examined whether the data allowed conclusions to be drawn based on frequency, intensity, time, and type. Although the heterogeneity of the protocols limits definitive conclusions, some patterns emerged. Studies reporting positive effects most used moderate-intensity aerobic training or combined aerobic and resistance training, performed at least three times per week for 8 to 12 weeks. Interventions of shorter duration or lower frequency produced less consistent results. Although these trends are preliminary and should be interpreted with caution, they may offer useful guidance for future research and program development.

Combined programs that integrated aerobic and resistance components, even in short 20 to 30 min sessions, showed positive effects, especially on inhibitory control and working memory. In terms of frequency and duration, interventions with at least three sessions per week for 6 weeks or more tended to report more consistent improvements. However, there was considerable variation in exercise types and formats (e.g., walking, cycling, and coordination training), making direct comparison difficult. Although limited by heterogeneity, these findings suggest that moderate-intensity, multi-component approaches may be particularly beneficial and should be further explored in future standardized protocols.

### 4.5. Interpretation of Null Findings

Among the included studies, several did not report significant improvements in executive function outcomes. A more in-depth analysis suggests that several factors could explain these null results. First, these studies were generally of lower methodological quality, as evidenced by PEDro scores below 6, with common limitations such as lack of follow-up, unclear randomization, or lack of blinding. Second, the interventions were often shorter in duration (≤6 weeks) or had a low training frequency (e.g., one session per week), which may not have been sufficient to achieve measurable cognitive benefits. Third, the use of less sensitive neuropsychological tests or those assessing specific executive subdomains (e.g., working memory only) may have limited the detection of significant changes. These factors, individually or in combination, may have contributed to the absence of positive results, and underscore the importance of adequate intervention dosage, sensitive assessment tools, and methodological rigor in future studies examining the cognitive effects of physical activity.

Despite the methodological heterogeneity across the included studies—such as differences in intervention duration, intensity, type of physical activity, and cognitive outcome measures—a qualitative comparison revealed some general patterns. Studies that implemented moderate-to-high intensity physical-activity interventions over longer durations (typically ≥8 weeks) more frequently reported improvements in executive functions, particularly in working memory and inhibition tasks. Additionally, interventions combining physical and cognitive components appeared to be more effective than unimodal ones. The variability in assessment tools (e.g., Stroop Test, Trail Making Test, and Digit Span) likely contributed to the differences in observed outcomes. Regarding effect sizes, only one study explicitly reported a Cohen’s d value (d = 0.25) [64], while the remaining articles did not provide standardized effect estimates. These limitations in reporting prevented formal quantitative synthesis but support the need for improved methodological consistency in future trials.

### 4.6. Limitations and Implications for Future Research

While the results appear promising, several limitations persist in the studies reviewed. The variability in intervention durations and the lack of methodological standardization make it challenging to define optimal protocols. Additional research is necessary to further explore the differences between exercise modalities and their specific effects. Designing experimental protocols with elderly participants is challenging due to difficulties in maintaining consistency throughout the intervention.

Additionally, several methodological limitations were identified in the studies reviewed. Many had small sample sizes, reducing statistical power. The lack of active control groups in most studies limits the interpretation of intervention effects. Risk of bias related to blinding, allocation concealment, and selective reporting were also noted in several trials, as reflected by the PEDro scores. Furthermore, few studies included follow-up assessments, limiting insights into the long-term efficacy of interventions. Future research should prioritize larger, methodologically rigorous trials, incorporate active control conditions, and include long-term follow-ups to assess the sustainability of cognitive improvements.

Despite the careful selection of keywords and search strings, some relevant studies were not identified in the initial search phase. This suggests that incorporating additional terms or keyword combinations could broaden and deepen the search, enabling a more comprehensive exploration of the relevant literature.

## 5. Conclusions

In the context of an aging population and the increasing interest in preventive strategies for cognitive health, this review aims to explore, through an analysis of the current scientific literature, the role of physical activity in maintaining and preserving executive functions—such as inhibition, working memory, planning, and cognitive flexibility—which are crucial for managing daily life and adapting to new or unforeseen situations. The specific aim was to determine not only whether physical activity can positively impact these functions during the aging process, but also which type of activity would be most effective for this purpose.

The reviewed scientific literature confirms that executive functions are among the first to decline with age but are also particularly sensitive to the positive effects of nonpharmacological interventions, with exercise standing out as one of the most effective. The findings emphasize that not all forms of physical activity yield the same effects. In addition to the well-established benefits of aerobic activity, there is growing evidence supporting practices that integrate motor, cognitive, and emotional components, such as dance, tai chi, Nordic walking, and mind–body interventions. These multidimensional approaches seem to work synergistically, stimulating brain activation, enhancing the efficiency of executive processes, and promoting overall well-being.

Considering these factors, the importance of promoting tailored physical-activity programs becomes clearer. These programs should be adaptable to the various stages of aging and address individual needs, enhancing existing resources and fostering cognitive plasticity. From a prevention and health promotion standpoint, physical activity emerges not only as a valuable tool for physical health but also as a genuine protective factor for the brain, supporting functional autonomy and quality of life, even in the later stages of adulthood.

Future research is likely to focus on longitudinal studies investigating the long-term effects of various exercise types on executive functions. Additionally, there is the potential for designing integrated interventions that combine physical activity, cognitive stimulation, and mindfulness practices; these could be implemented in educational and community settings. Another promising direction is to assess the preventive effectiveness of such educational interventions when introduced as early as middle age. The aim would be to strengthen cognitive resources early and slow cognitive decline before it becomes clinically significant.

## Figures and Tables

**Table 1:** Summary of the literature analysis.

Authors	Participants	Average Age	Objective	Methodology	Type of Physical Activity	Duration	Evaluation Tools	Results	Pedro Score
Byun, et al. [67]	81	68	To evaluate the effects of mild exercise on executive function and cortical activation.	Participants were randomly assigned to an exercise group or a control group.	Cycloergometer.	3 months, (3 sessions of 30–50 min each)	Stroop test	Long-term moderate exercise improves executive performance in middle-aged and older adults.	5
Nouchi, et al. [63]	59	62.29	Investigating the effects of a single 30 min exercise training session with combined exercises on cognition and mood in middle-aged and elderly women.	Participants were randomly assigned to two groups: a combined exercise group and a control group without exercises.	The exercise included aerobic, strength-based, and stretching exercises.	Single session lasting 30 min	Wechsler Adult Intelligence Scale; Stroop Task; Reverse Stroop Task; Verbal fluency task	Compared to the control group, the combined exercise group showed significant improvement in inhibition.	7
Pellegrini-Laplagne, et al. [68]	35	63.9	To evaluate the impact of the simultaneous combination of physical and cognitive training on specific cognitive domains and compare these methods with physical or cognitive training alone.	Participants were randomly assigned to three groups: physical training, cognitive training, and combined training.	Cycloergometer.	12-week program with two weekly 30 min sessions	Montreal (MOCA); 2-back; Stroop; Trail Making; Rey words; Digit Symbol Substitution; Baroreflex Sensitivity	Simultaneous physical and cognitive workouts resulted in significant improvements in cognitive function, compared to physical or cognitive training alone. Notable benefits were observed in divided attention, executive control, and working memory.	5
Lebeau, et al. [25]	62	75.58	To investigate the effect of an acute exercise session on executive function, balance, and life skills among healthy elderly participants.	Participants were randomly assigned to two groups: the exercise group (performed 20 min of moderate-intensity cycling) and the control group (watched a video on the importance of exercise and rest for well-being).	Cycloergometer.	Single 20 min session	Mini Mental State Examination; Geriatric Depression Scale; Tests for color blindness; Stroop Test	The 20 min cycling session had immediate positive effects on balance among healthy older adults. However, it did not lead to significant changes in executive function.	5
Martini, et al. [62]	48	62.1	To explore the effects of 20 min of moderate-intensity aerobic exercise on cognitive functions.	Participants were divided into two groups: an experimental group, which performed 20 min of cycling, and a control group, which remained inactive.	Cycloergometer. Moderate intensity.	Single 20 min session	Affective Go/No-Go; Simple Reaction Time; Spatial Working Memory	Acute moderate-intensity exercise can improve inhibitory control functions and enhance certain aspects of working memory.	5
Díaz-García, et al. [66]	24	71.42	To examine the effects of Brain Endurance Training (BET) on cognitive and physical performance in both “fresh” and “fatigued” conditions among older adults.	Participants were divided into three groups: a Brain Endurance Training group (which performed cognitive tasks before physical exercise); a physical training group (performed only physical exercise); and a control group, which did not engage in training.	Treadmill walking for 25 min, or endurance training at moderate intensity.	The BET group and the physical training group performed three sessions of 45 min per week for eight weeks	Brief Stroop Task; Brief Psychomotor Vigilance Task	Both the BET and physical training groups showed superior cognitive and physical performance, in both fresh and fatigued states, at mid- and post-intervention compared to the control group.	5
Pereira, et al. [65]	33	83.94	To explore the effect of a personalized exercise program on cognitive function in the elderly, with the goal of counteracting age-related cognitive decline.	Participants were divided into two groups: an intervention group, which followed a personalized physical exercise program, and a control group, which continued their usual daily activities.	Walking for at least 10 min on flat or sloped ground, joint mobilization exercises, respiratory coordination, and balance and coordination exercises. Moderate intensity.	3 months	Cambridge Neuropsychological Test Automated Battery	Personalized exercise was found to be an effective strategy for counteracting cognitive decline associated with aging. No significant changes in cognitive function were observed in the control group.	4
Gervasi, et al. [69]	61	72.84	To investigate the relationship between physical activity, cognitive function, and social well-being, demonstrating how structured physical and cognitive activity programs can improve quality of life in the elderly.	Participants were randomly assigned to two groups. Both groups followed the same program, one that combined adapted physical activity (APA) with cognitive and socio-emotional training.	Floor exercises in a gym setting, aimed at enhancing endurance, strength, balance, coordination, and flexibility.	Moderate combined PA, 2x/week, 12 weeks, 60 min	Short Form Health Survey; Trail Making Test; Serial Repetition Bisyllabic Words Test	Integrated physical and cognitive activity programs may slow age-related cognitive decline and improve attention and memory, thus enhancing overall well-being in the elderly.	5
Lebeau, et al. [64]	71	66.39	To examine the effects of a single episode of aerobic exercise on executive function and driving performance in elderly adults.	Participants were randomly assigned to one of two groups: an exercise group (performed aerobic exercise using a cycloergometer) and a control group (watched a video providing driving-related information).	Cycloergometer.	Single moderate aerobic session lasting 25 min	Trail Making Test; Stroop Test; Useful Field of View Test	The exercise group demonstrated improvements in executive functions, particularly in flexibility and inhibition, compared to the control group. However, no significant effects were observed on driving performance.	6

## Data Availability

No new data were created or analyzed in this study.

## Supplementary Materials

The following supporting information can be downloaded at: https://www.mdpi.com/article/10.3390/brainsci15070703/s1, Table S1: Quantitative details of the physical activity interventions across included studies, including frequency, duration, intensity (expressed as %HRmax, HRR, or RPE), and type of exercise performed; Table S2: Basic characteristics of participants. Overview of participants’ health status, previous physical activity levels, and baseline cognitive scores; Table S3: Type and difficulty of cognitive training. Description of the cognitive training tasks used in each study, specifying task types, difficulty levels, and the targeted cognitive domains.
