# Supplementary material for: The Effect of Physical Activity on Executive Functions in the Elderly Population: A Systematic Review of Randomized Controlled Trials
Source: Brain Sci. 2025 Jun 30;15(7):703. doi: 10.3390/brainsci15070703 (PMC12293948; doi:10.3390/brainsci15070703)
Supplement: Supplementary file 1 [file brainsci-15-00703-s001.zip › brainsci-3697223-supplementary.pdf]

Table S1. Quantitative details of the physical activity interventions across included studies, including frequency, duration, intensity (expressed as %HRmax, HRR, or RPE), and type of exercise performed.

AUTHORS	TYPE OF PHYSICAL ACTIVITY	EXERCISE INTENSITY	HEART RATE RANGES	RPE SCORE	FREQUENCY AND DURATION
Byun, et al. [67]	Cycloergometer	Very light	~59-61% HRmax	Not specified	3 months, (3 sessions of 30-50 minutes each)
Nouchi, et al. [63]	The exercise included aerobic, strength, and stretching exercises	Moderate	71% HRmax	Not specified	Single session lasting 30 minutes
Pellegrini-Laplagne, et al. [68]	Cycloergometer	Moderate	60% tHRmax	Not specified	12-week program with two weekly 30-minute sessions
Lebeau, et al. [25]	Cycloergometer	Moderate	57-63% HRR	Not specified	Single 20-minute session
Martini, et al. [62]	Cycloergometer	Moderate	60% HRmax ± 5 bpm	Not specified	Single 20-minute session
Díaz-García, et al. [66]	25 minutes of treadmill walking or endurance training	Moderate	Not specified	7-8 Borg	The BET group and the physical training one performed: three sessions of 45 minutes per week for eight weeks
Pereira, et al. [65]	Walking for at least 10 minutes on flat or sloped ground, joint mobilization exercises, respiratory coordination, and balance and coordination exercises	Moderate	Not specified	Not specified	3 months
Gervasi, et al. [69]	Floor exercises in a gym setting aimed at enhancing endurance, strength, balance, coordination, and flexibility	Moderate	Not specified	Not specified	Moderate combined PA, 2 days/week, 12 weeks, 60 minutes
Lebeau, et al. [64]	Cycloergometer	Moderate	57–63% HRR	Not specified	Single moderate aerobic session lasting 25 minutes

Table S2. Basic characteristics of participants. Overview of participants' health status, previous physical activity levels, and baseline cognitive scores,

AUTHORS	HEALTH STATUS	PRIOR PHYSICAL ACTIVITY LEVELS	BASELINE COGNITIVE SCORES
Gervasi, et al. [69]	Low to moderate scores on the SF-36, particularly in the cognitive domain (MCS=33.05)	Mostly sedentary. 45.9% practice sports occasionally.e	TMT-A: 197.98 sec<br>TMT-B: 624.30 sec→ reduced cognitive attention and flexibility<br>SRBWT: 1.98 impaired short-term memory
Lebeau, et al. [64]	Healthy participants	Not specified	Cognitive tests (Stroop test and TMT) with a baseline that is not numerically detailed but used as a reference for the effects of exercise.
Pereira, et al. [65]	Independent participants with no known neurological disorders	Not specified	CANTAB with numerical scores not reported in detail at baseline, but used as a reference for post-intervention
Díaz-García, et al. [66]	Healthy participants	Sedentary	Stroop test and PVT-B; numerical values not specified
Martini, et al. [62]	Healthy participants	Not specified	AGN, SWM, SRT, and backward counting before surgery; baseline values not reported
Lebeau, et al. [25]	Healthy participants	Assessed with MLTPAQ variable levels, no exclusion for sedentary lifestyle	Stroop, medication task administered before intervention, numerical values not reported
Pellegrini-Laplagne, et al. [68]	Healthy participants	Not specified	Average MOCA = 27.7 ± 1.6<br>Stroop, TMT, Rey words test, and N-back administered, but baseline values not reported numerically
Nouchi, et al. [63]	Healthy participants	Active participants	Middle-aged women (Cd=90.21; rST=36.86; ST=36.71; LFT=10.43; WM=11.36; D-CAT=201.50)<br>Older women (Cd=71.67; rST=25.80; ST=24.60; LFT=9.00; WM=8.73; D-CAT=171.13)
Byun, et al. [67]	Healthy participants	Sedentary participants	ST: 349.1ms → exercise group<br>325.7 ms → control group

SF= Short Form Health Survey
MCS= Mental Component Summary
TMT= Trail Making Test
SRBWT= Serial Repetition Bi-syllabic Words Test
CANTAB= Cambridge Neuropsychological Test Automated Battery
PVT-B= Brief Psychomotor Vigilance Task
AGN= Affective Go/No-Go
SWM= Spatial Working Memory
SRT= Simple Reaction Time
MLTPAQ= Minnesota Leisure-Time Physical Activity Questionnaire
MOCA= Montreal Cognitive Assessment
Cd= digit symbol coding
rST= reverse Stroop task
ST= Stroop task
LFT=letter fluency task
WM= Working Memory
D-CAT=Digit Cancellation Task
ST=Stroop Test

Table S3. Type and difficulty of cognitive training. Description of the cognitive training tasks used in each study, specifying task types, difficulty levels, and the targeted cognitive domains.

AUTHORS	TYPES OF COGNITIVE TASKS	DIFFICULTY LEVELS	COGNITIVE DOMAINS TARGETED
Gervasi, et al. [69]	TMT A, TMT B, SRBWT	TMT-B is more complex than TMT-A.<br>SRBWT: progressive length of verbal strings to be repeated	TMT: attention, cognitive flexibility, executive functions<br>SRBWT: short-term verbal memory
Lebeau, et al. [64]	ST, TMT A, TMT B, UFOV	ST: includes congruent and incongruent conditions with reaction time accuracy measurement<br>TMT-B: letter-number alternation<br>UFOV: includes visual distractors, increasing selective complexity	ST: inhibition, selective attention, cognitive shifting<br>TMT: processing speed, cognitive flexibility<br>UFOV: divided attention, selective attention, visual processing speed
Pereira, et al. [65]	MOT, SWM, PAL	MOT: basic motor assessment<br>SWM: increasing difficulty<br>PAL: up to 8 patterns to be correctly matched, with a maximum of 10 attempts for each stage	MOT: motor skills and sensorimotor processes<br>SWM: spatial working memory, search strategy, executive functions<br>PAL: associative visuospatial memory, visual learning
Díaz-García, et al. [66]	Brief Stroop Task, PVT-B	Stroop: only inconsistent evidence → high inhibitory load<br>PVT-B: stimuli at variable intervals, requires sustained vigilance	ST: response inhibition, executive control<br>PVT-B: sustained attention, processing speed
Martini, et al. [62]	AGN, SWM, SRT, Backward Counting Task	AGN: 180 words with congruent/incongruent blocks and rapid response to emotional stimuli<br>SWM: increasing difficulty, requires strategy and spatial memory<br>SRT: simple, with variable times between stimuli<br>Backward Counting: requires sustained attention	AGN: response inhibition, executive control<br>SWM: spatial working memory, executive strategies<br>SRT: processing speed, motor responsiveness<br>Backward Counting Task: working memory, sustained attention
Lebeau, et al. [25]	ST, Medication Task	ST: includes inconsistent evidence → requires inhibition and selective attention	ST: response inhibition, selective attention<br>Medication Task: complex executive functions, working memory, planning

		Medication Task: requires working memory and planning	
Pellegrini-Laplagne, et al. [68]	Modified Stroop Task, TMT-A, TMT-B, Rey Word Test, DSST, N-back	ST: blocks with congruent, incongruent, and switching conditions<br>TMT-B: complex, alternating numbers and letters	ST, TMT-B: inhibition, cognitive flexibility, executive control<br>DSST, TMT-A: processing speed, motor reactivity<br>N-back: working memory, sustained attention<br>Rey: verbal learning, long-term memory
Nouchi, et al. [63]	Cd, ST, rST,n LFT, Working Memory Updating Task, D-CAT	Cd: associate symbols with numbers<br>ST/Rst: incongruent trials, requiring inhibition<br>LFT: word generation in 1 min<br>Working Memory Updating Task: remember the last 3 or 4 digits of increasing lists<br>D-CAT: delete 3 target digits in 1 min on sheets with 600 numbers	Cd: processing speed<br>ST, rST: inhibition, executive control<br>LFT: cognitive flexibility<br>Working Memory Updating Task: working memory, updating<br>D-CAT: sustained attention, visual accuracy
Byun, et al. [67]	CWST	Inconsistent CWST: require cognitive inhibition<br>Neutral CWST: colored letters without semantic conflict	CWST: response inhibition, executive control, selective attention

TMT= Trail Making Test
SRBWT= Serial Bi-syllabic Words Test
ST= Stroop task
UFOV =Useful Field of View
MOT= Motor Screening Task
SWM= Spatial Working Memory
PAL= Paired Associates Learning
PVT-B =Brief Psychomotor Vigilance Task
AGN =Affective Go/No-Go
SRT=Simple Reaction Time
DSST =Digit Symbol Substitution Test

Cd= digit symbol coding
rST= reverse Stroop task
LFT=letter fluency task
D-CAT=Digit Cancellation Task
CWST=Color- Word Matching Stroop Task
